# Acute Pancreatitis Due to Disseminated Varicella Zoster Infection in an Individual with Newly Diagnosed Human Immunodeficiency Virus

**DOI:** 10.7759/cureus.7027

**Published:** 2020-02-18

**Authors:** Sasmit Roy, Subhasish Bose, Ramesh K Pandey, Srikanth Naramala, Muhammad Rajib Hossain

**Affiliations:** 1 Nephrology, University of Virginia, Lynchburg, USA; 2 Nephrology / Internal Medicine, Lynchburg General Hospital, Lynchburg, USA; 3 Oncologic Emergency, MD Anderson Cancer Center, Houston, USA; 4 Rheumatology, Adventist Medical Center, Hanford, USA; 5 Hospital Medicine, Geisinger Medical Center, Danville, USA

**Keywords:** disseminated varicella, hiv, acute pancreatitis, varicella zoster, immunocompromised hosts, pneumocystis carinii pneumonia

## Abstract

Varicella-zoster virus (VZV) infection is generally considered as a benign and self-limiting disease. However, individuals with VZV infection can have disseminated to various organs leading to serious complications, particularly in adults. This pattern is more prevalent in immunosuppressed individuals. Disseminated varicella is historically known to involve the central nervous system (CNS), liver, and lungs. However, dissemination of varicella to the pancreas and subsequently causing acute pancreatitis has been rarely reported.

We present a case of disseminated varicella infection in a newly diagnosed human immunodeficiency virus (HIV) patient causing acute pancreatitis at initial disease presentation and subsequently leading to multi organ dysfunction.

A 42-year-old African American female who was initially being treated for Pneumocystis carinii pneumonia (PCP) at an inner-city hospital developed severe epigastric pain radiating to back along with nausea on day 2 of admission. Physical findings revealed tachycardia, epigastric tenderness and newly formed vesicular rash involving the neck and face classical of varicella infection. Skin biopsy and serum sample confirmed varicella infection by VZV polymerase chain reaction (PCR) test. Labs revealed elevated lipase, amylase at a level diagnostic of acute pancreatitis. The patient had no other risk factors for pancreatitis. She was started on intravenous Acyclovir and intravenous hydration with isotonic normal saline. She was managed conservatively for other systemic complications. Pancreatitis resolved after five days of clinical presentation. She completed two weeks of Acyclovir, her condition steadily improved and she was successfully discharged home with no further recurrence.

Acute pancreatitis is a rare infectious association of disseminated varicella infection. Clinicians should always be mindful of this infectious etiology as one of the rare differentials for acute pancreatitis as this is a treatable cause and could prevent morbidity, mortality associated with this condition if treated timely.

## Introduction

Varicella-zoster virus (VZV), a member of the herpes zoster family, is notorious for causing varicella infection among young children while causing herpes zoster infection in adults with immunocompromised status. The VZV lies dormant in the dorsal root ganglia of the sensory nerves after initial primary infection. This later can reactivate with unilateral spread along a particular dermatome leading to herpes zoster infection. The characteristic varicella rash is a pruritic one appearing in successive crops over several days. Initially preceded by a prodrome of fever, malaise, or pharyngitis, the rash appears after 24 hours. The lesions normally begin as pruritic macules that can rapidly transform to papules and subsequently to the characteristic vesicles. This rash covers single/multiple dermatomes and persists for average of three to five days. The most frequent site of reactivation is the ophthalmic division of the trigeminal nerve, which can lead to the involvement of the thoracic nerves and the eyes. In the absence of a typical rash, herpes zoster is confirmed by a virology laboratory through testing of VZV polymerase chain reaction (PCR) [1, 2]. A secondary bacterial infection is among the commonest of the complications. Other serious complications include hepatitis, pneumonia, encephalitis, disseminated intravascular coagulopathy, myelitis, retinitis, hemiparesis, which are more common in immunocompromised patients, such as transplant recipients and patients with acquired immune deficiency syndrome (AIDS) [3]. The occurrence of acute pancreatitis due to disseminated VZV infection is extremely rare and a very few cases are only reported. We present a rare case of a female with incidentally detected HIV, who end up having acute pancreatitis with multi organ involvement from disseminated varicella-zoster infection.

## Case presentation

A 42-year-old African American female who recently emigrated from the Caribbean Islands initially presented with non-resolving productive cough and dyspnea for two weeks. She had no significant past medical history, no history of medication use, no history of surgery, or recent prolonged immobilization. Admission vitals were: blood pressure (BP) 110/75, temperature 98.2 F, respiratory rate 18/min, sPO2 95% room air, tachycardia with 110-120 beats/min. She was anxious on presentation. Rest of the physical exam including cardiovascular, gastrointestinal, and respiratory system findings, were unremarkable.

**Figure 1:**
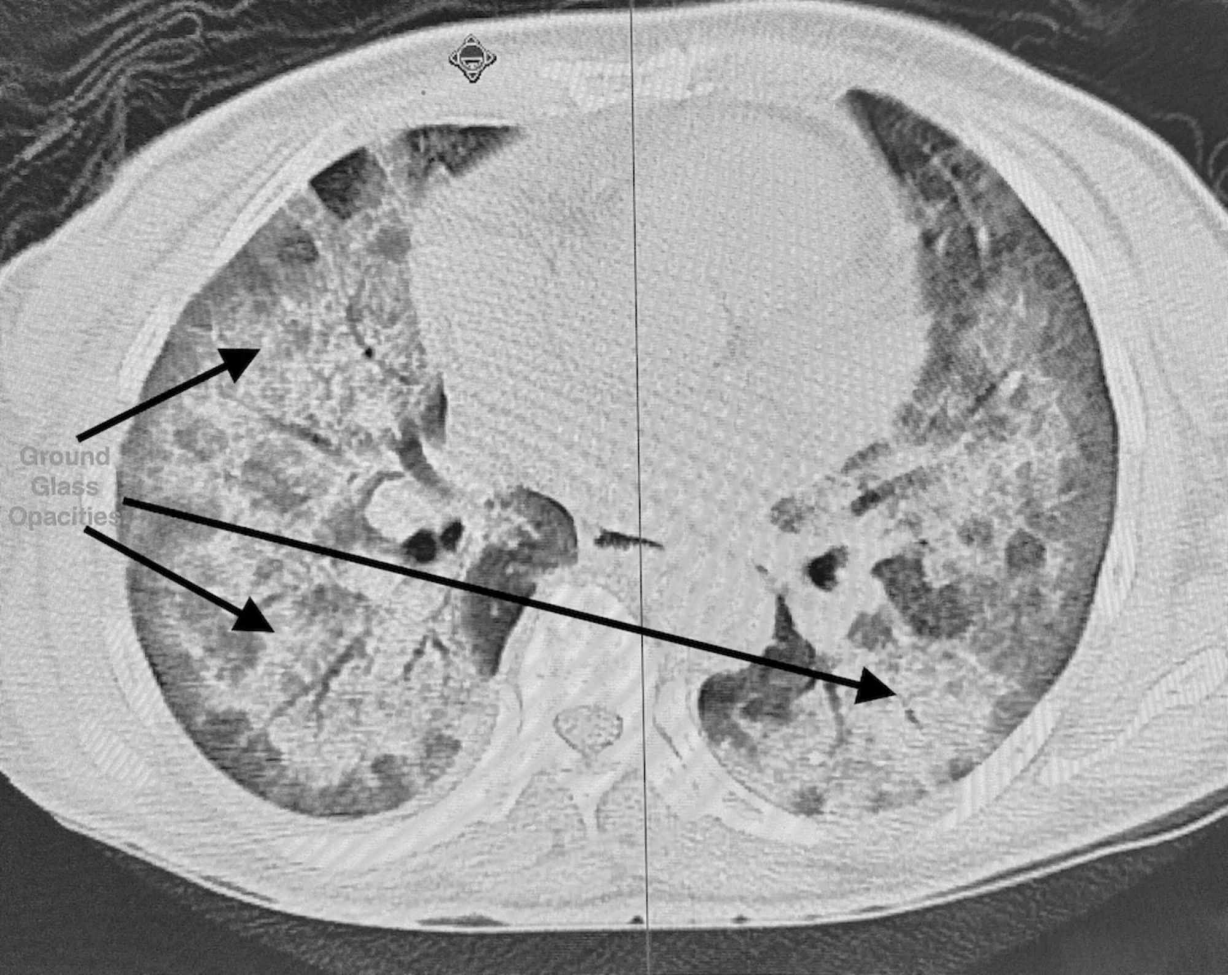
Bilateral ground glass opacities

**Figure 2:**
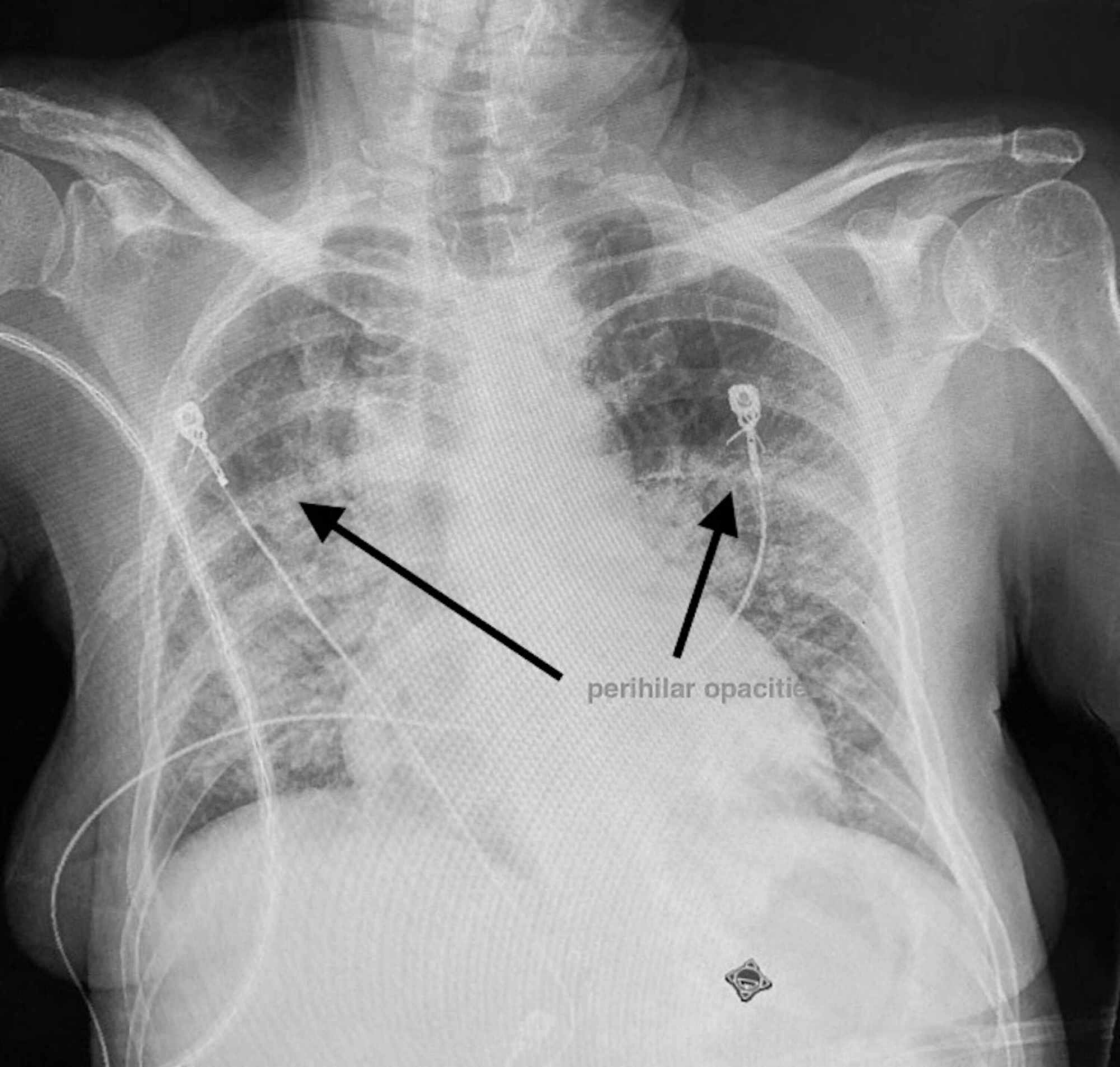
Bilateral perihilar opacities classic of Pneumocystis carinii pneumonia (PCP)

Initial investigations revealed the following: White blood cell count 9.9 x 10^9^/L (ref 4-11 x 10^9^/L), hemoglobin 11.7 gm/dl (reference 13-15 gm/dl), hematocrit 36.8% (reference range 39-48%), platelets 304,000/ml (reference 15,000 to 450,000/ml); arterial blood gas analysis suggested hypoxia, pO2 being 72% and had high A-a gradient of 37 mm Hg (normal 5-10 mm Hg); lactate dehydrogenase (LDH) was elevated to 508 unit/L (reference range 140-280 units/L). Electrocardiogram (EKG) showed sinus tachycardia. CT angiogram chest did not show any pulmonary embolus but showed diffuse ground glass lung opacities with hilar lymphadenopathy (Figure 1). She underwent voluntary human immunodeficiency virus (HIV) testing as per New York State law, and the test came back reactive with CD4 count 46 cells/micro litre (reference range 500-1500 cells/micro litre) and HIV viral load 810231 copies/ml (reference range 40-50 copies/ml). The urine legionella antigen was negative. Treatment was initiated with oral Bactrim DS and Prednisone (for suspected Pneumocystis carinii pneumonia [PCP]), and further investigation by bronchoscopy specimen was positive for Pneumocystis carinii PCR confirming PCP. The patient improved clinically and was discharged home with continuation therapy of Bactrim DS and outpatient follow-up instructions. However, she was re-admitted to the hospital within a week of discharge with worsening dyspnea and cough with expectorations. Vitals showed sinus tachycardia with heart rate 110/min. Other vitals like BP, temperature, respiratory rate were normal. Chest X-ray on admission showed perihilar opacities unchanged from previous admission (Figure 2). Physical examination revealed rales bilaterally. The rest of examinations, including gastrointestinal, neurological, cardiovascular system, were normal. Treatment was initiated with broad spectrum antibiotics Vancomycin and Meropenem intravenous (IV) in view of possible superadded bacterial infection. On day 2 of admission, she complained of severe epigastric pain radiating to back and also was noted to have pruritic macular rashes over face and neck. As initial suspicion was Varicella Zoster (VZ) infection vs. drug-induced rash, Bactrim was stopped, and she was started on Valacyclovir orally. Serum lipase and amylase were elevated at 160 IU/dl (reference range less than 50 IU/dl) and 133 IU/dl (reference range less than 110 IU/dl) respectively. The rest of the labs, including blood urea nitrogen, creatinine, calcium, sodium, potassium, phosphorus, were normal. She was also started on IV hydration with isotonic normal saline and pain management with opioids for pancreatitis. Her clinical condition continued to worsen gradually, and the rash became more generalized with pruritic vesicular-papular eruptions. She developed a low-grade fever and became hypotension - BP 70/40 (she required inotropic support with norepinephrine for three days). Other systemic organs were also involved, as evident by laboratory abnormalities. Over the course of hospital stay, her labs got worsened significantly - platelet counts dropped remarkably (125-66-30---26---24); Lipase/amylase continued to increase (160/133-379/663---254/491); liver functions worsened with aspartate aminotransferase (AST)/alanine aminotransferase (ALT) gradually increased (180/128---389/227---837/501) (reference range < 40 for both); LDH was high (2438---2565-1308); lactic acid increased (3.7-2.4) [reference range < 2 mmol/L]. Given the worsening clinical status, she was switched to Acyclovir 500 mg IV. Her blood culture and urine culture sent on admission day returned negative for any bacterial infection; four days later, her VZ virus DNA PCR from skin blister and serum both reported positive confirming active VZV infection.

Ultrasound abdomen did not reveal any gallstones. Echocardiogram did not suggest any source of septic emboli.

Her clinical condition improved gradually with near normalization of labs. She completed 14 days course of antimicrobials for disseminated varicella. Eventually, she was started on an anti-retroviral drug Stribild (Dolutegravir/Lamivudine) daily along with a prophylactic dose of Dapsone (for PCP). Post treatment completion, oral Valacyclovir was initiated for viral suppression. After the acute stay in hospital for three weeks, she recovered well and was followed up regularly in the clinic with no further recurrence in six months of follow-up.

## Discussion

Acute pancreatitis is an inflammatory condition of the pancreas, also known to involve the peri-pancreatic tissues. The two commonest etiologies of acute pancreatitis in adults are common bile duct stones and alcohol abuse, accounting for 36% and 38%, respectively. Other common causes include surgery, various metabolic or autoimmune conditions, toxins, drugs, as well as infections (from bacteria, viruses, mycoplasma, and parasites) [4].

Till now, only a few cases of herpes zoster-associated acute pancreatitis have been reported; among them, most involve immunocompromised individuals, such as those in intensive care, AIDS patients, or receiving long-term immunosuppression, such as recipients of stem cell, renal or liver transplants [5-9]. There were only a handful of studies which reported acute pancreatitis associated with VZV in children with chickenpox and one study reported an elderly patient with herpes zoster suffering from systemic complications, including pancreatitis and encephalitis [10-12, 13]. Interestingly, the last case referred to was the first report of acute pancreatitis in an immunocompetent adult without any significant comorbidity. Although the mechanism of pancreatitis with herpes zoster is still unknown, VZV may remain latent in posterior sensory nerve roots that contain fibers from both skin and abdominal viscera, including the pancreas. Till now, with no definitive mechanism discovered, the common theory is that VZV remains latent in posterior sensory nerve roots which contain fibers from both abdominal viscera, including the pancreas and also from the skin. It is speculated that VZV might mediate this injury through damage of the pancreatic acinar cell membrane causing leakage of intracellular pancreatic enzymes. Another mechanism hypothesized is that the pancreas own immune response may mediate the cytopathic effect.

Immune-compromised patients are at increased risk of VZV reactivation, including transplant recipients, patients receiving selected immune modulator therapies, and HIV-infected patients [14-16]. These individuals also present with higher chances of complications from the disease (e.g., disseminated disease, ocular involvement) [3,4]. The incidence of herpes zoster has remained greater in HIV-infected individuals despite the wide use of potent antiretroviral therapy (ART).

Immunocompromised individuals like HIV-infected individuals with advanced disease and transplant recipients are at increased risk for developing complicated herpes zoster infections [16,17]. These complications include cutaneous dissemination and visceral end organ involvement. Cutaneous dissemination often presents with multiple vesicular skin lesions in a generalized distribution affecting a number of distinct dermatomes. Visceral organ involvement due to herpes zoster may present in a fulminant manner with a rapid evolving syndrome like hepatitis, pneumonia, or encephalitis and may occasionally develop in the absence of coincident rash [18].

As mentioned earlier, immunocompromised persons who get varicella are at risk of developing visceral dissemination (VZV infection of internal organs) rapidly, culminating to hepatitis, encephalitis, pneumonia, and disseminated intravascular coagulopathy. They can have an atypical varicella rash with more and atypical lesions, as well as can have a prolonged course lasting longer than those who are not immunocompromised. New evolving lesions may continue to develop for more than a week, may appear on atypical places like the palms and soles, and may be hemorrhagic in nature [2]. What causes reactivation of VZV is still a mystery begging to be solved. It is presumed that the virus remains latent in the dorsal root ganglia after the initial benign presentation as chickenpox and get reactivated later. During active herpes zoster, histopathology examination of representative dorsal root ganglia demonstrates edema, hemorrhage, and lymphocytic infiltration. Active replication of VZV in other organs, such as in the lung or the brain, can occur during either chickenpox or herpes zoster but is uncommon in the immunocompetent host.

Like chickenpox, herpes zoster is more severe in immunocompromised than immunocompetent individuals. Lesions continue to evolve for more than a week, and scabbing is not complete in most cases until three weeks into the illness. Among patients with cutaneous dissemination, the risks of dreaded complications like meningoencephalitis, pneumonitis, hepatitis, and other serious complications are increased by 5-10%. However, disseminated zoster is rarely fatal, even in immunocompromised individuals.

Antiviral therapy should be initiated in all immunocompromised patients with herpes zoster, even if they present after 72 hours. Rapid initiation of therapy is particularly critical in the severely immunocompromised patient. The nucleoside analogues acyclovir, valacyclovir, and famciclovir are the preferred antivirals for the treatment of acute herpes zoster infection. Oral antiviral therapy is usually sufficient for the initial treatment of uncomplicated herpes zoster, unless the patient has evidence of complicated disease (e.g., acute retinal necrosis, encephalitis).

Very few cases of acute pancreatitis after disseminated varicella have been reported so far [6,19].

## Conclusions

Disseminated varicella infection has been notorious for involving multi system organs like central nervous system (CNS), lungs, hematological system mainly disseminated intravascular coagulopathy, and liver. The incidence of acute pancreatitis following disseminated varicella has rarely been reported. Asymptomatic pancreatitis may well be associated with VZV and has not been well documented for so far. Clinicians should keep a high index of suspicion of pancreatitis for such patients and intervene early to prevent complications.
